# Play Therapy As Effective Options for School-Age Children With Emotional and Behavioral Problems: A Case Series

**DOI:** 10.7759/cureus.40093

**Published:** 2023-06-07

**Authors:** Nihit Gupta, Ridhimaa Chaudhary, Mayank Gupta, Linh-Han Ikehara, Faiza Zubiar, Jayakrishna S Madabushi

**Affiliations:** 1 Psychiatry, Dayton Children’s Hospital, Dayton, USA; 2 Psychiatry and Behavioral Sciences, Santokba Durlabhji Hospital, Jaipur, IND; 3 Psychiatry and Behavioral Sciences, Southwood Psychiatric Hospital, Pittsburgh, USA; 4 Psychology, Dayton Children's Hospital, Dayton, USA; 5 Psychiatry, The Trenton Psychiatric Hospital, Trenton, USA; 6 Psychiatry, Alabama College of Osteopathic Medicine, Birmingham, USA

**Keywords:** development, cognitive behavioural therapy (cbt), child and adolescent psychiatry, emotional and behavioral problems, play therapy

## Abstract

As a type of psychotherapy, play therapy entails using play and creative activities as a means to help children express their thoughts and emotions, and to work through their challenges. A wide range of issues can be addressed through play therapy, including behavioral issues, anxiety, depression, trauma, and difficulties in relationships. Through this case report, we aim to discuss the history and evolution of play therapy concepts. We will review the core principles of child-centered therapy (CCT), non-directive child-centered play (NDCCP), and cognitive behavior play therapy. We will discuss clinically helpful approaches and the evidence supporting the efficacy of play therapy in anxiety, depression, trauma, and other behavioral difficulties in children.

## Introduction

Play therapy (PT) started to materialize in the early 20th century when theorists started viewing it as a medium to gain insight into the inner emotional world of a child. As play is a child’s natural language, developmentally, few children achieve the cognition and vocabulary to express their feelings. The history of PT dates back many years. It is difficult to ignore the fact that the first obvious reference can be traced back to Sigmund Freud in 1909 with the example of “Little Hans,” a five-year-old boy manifesting phobic symptoms [1], but the approach was psychodynamic with a significant interpretation. Furthermore, Freud had never met Hans in person. This had a close resemblance to “Filial Therapy” [2]. Filial therapy is time-limited therapy with a focus on teaching parents effective ways of working and playing with the child. Klein, in 1935 used play as a medium for free association [3] followed by Levy in 1938 with the creation of Release Therapy, a structured approach [4]. Anna Freud used play to build rapport with children for facilitating therapy in 1946 [5]. Hambridge in 1955 expanded on this and created structured PT [6]. It was in 1951 when Carl Rogers introduced Nondirective PT (NDT) [7], also called person-centered PT. Axline in 1947 expanded his work on NDT [8]. Landreth further expanded on Axline’s work [9,10]. The core principle of child-centered therapy (CCT) or non-directive child-centered play (NDCCP) is based on the person-centered approach of Carl Rogers [7]. He believed clients find their solutions to achieve self-actualization with the help of a nurturing therapeutic relationship. He propagated that a therapeutic relationship involving empathy, unconditional positive regard, and congruence with active listening and reflection can bring about change. In 1982, the Association for PT (APT) was launched. This led to the development of professional standards and the advancement of the field [11].

Piaget highlighted that in the first decade of a child’s development, s/he lacks meaningful expressions and comprehension of complex situations, motives, and feelings due to a lack of abstract thinking, he also noted that in the-operational phase, i.e., 2-7 years of age, the child starts with symbolic play [12]. PT is a space provided where a child plays out his or her feelings and problems just as in adult therapy, an individual “talks out” his or her difficulties [8]. From a neuroscientist’s perspective, the effects of trauma reside in the nonverbal areas of the brain (the hippocampus, amygdala, thalamus, and brain stem), while the ability to communicate the adverse effects of it resides in the frontal lobe. The symbolic and role-play activities associated with PT help move the traumatic experiences from the nonverbal brain to the frontal lobe [13].

One vital ingredient in the therapeutic relationship is a corrective relationship, i.e., therapist’s response is different from the child’s anticipated reaction through play. When the therapist's response alters with the anticipated reaction, the transformation of core beliefs takes place, in children with sub-average cognitive abilities it takes the form of supportive scaffolding [10]. Landreth also highlighted the importance of understanding the child’s world and perspective [14]. PT takes place in a play that room playroom which is equipped with a selection of play materials, these are specifically chosen toys that encourage the child to express his or her feelings and develop new ways of being or healthier behaviors. The interaction with the toys present presents a child's symbolic world. The therapist gets acquainted with specific thoughts and emotions that a child would otherwise find difficult or impossible to verbally express.

Usually, the toys used include doll houses, sandbox, miniature figurines, art material, stuffed animals, construction toys or legos, costumes, dolls, puppets, indoor play material, board games, other tools, methods like storybooks, clay, music, dance, drama, role play, and creative visualization can be incorporated. The material helps the child to tell his or her story.

At the beginning of the journey of PT, the child gets to explore the playroom to get comfortable in the space. This is followed by the therapist working as a facilitator wherever the child needs encouragement through the introduction of tools through which the child can narrate his story or *overcome resistance* [15]. Children often present as angry, upset, frightened, withdrawn, shy, or inhibited during therapy and accordingly mount resistance as an unconscious process to strategically deflect the topic of discussion, this could be manifested when the topic of discussion is overwhelming/painful. The therapist’s observation of the sequences and play themes helps in gaining a deeper insight into the child’s inner world, the traumatic experiences that have had an impact on the child will show up in the play, like, a child who has moved to a new home due to parents' separation will show this by repeatedly changing houses using doll house. The doll house provides a mechanism for the child to reenact family dynamics as it pertains to his feelings of closeness to those in the households of his parents as well as his transition from one household to the other. Using creates a safe, free, and protected space, this further gives the child the opportunity to work through deeper emotional wounds, fears, and experiences [16]. PT facilitates healing from past traumatic or stressful events. These experiences may get stuck in the mind or even out of a child’s awareness, this can result in emotional and behavioral problems that adults observe in a child. PT aims at raising child’s awareness by making sense of stressful or traumatic experiences and assimilating them into their comprehension. The child gains perspective and rehearses new ways of being and resolves the problem [17]. We would like to present five cases where PT was beneficial among children.

## Case presentation

Case 1

A nine-year-old female second-grade student was referred to a child psychologist by her pediatrician for pseudoseizures. The child was verbose and engaged in conversation with the clinicians. She shared that she wishes to achieve the highest score in her academics. During the assessment, her parents reported that the child was born full-term with an immediate birth cry, and she had no developmental delays. The child was taking treatment for seizures until her pediatrician found out that the episodes were taking place only in school.

On further discussion, it was found out that the child had started presenting with the concern of seizures one year and this started after she moved in with her grandparents. Father shared that the home environment has conflicts as the grandmother is aggressive and the child is scared of her. The child was facing difficulty with academic performance. The child's verbal intelligence was assessed using Malins Intelligence Scale For Indian Children (MISIC) and scored average intellectual functioning. She was diagnosed with a learning disability and her mother was a teacher which had an impact on her academic expectations. Parents were provided with psychoeducation regarding her treatment strategies, and NDT was commenced. The child received four sessions in a month. NDT was chosen for her as she appeared to be motivated to do better but had difficulties communicating her feelings. The child expressed herself through miniature dolls and often presented her classroom and home environment in play. In one of the sessions child revealed through her play that her mother scolds her for her studies and calls her “useless.” This was brought to the notice of parents and academic stress was further reduced with special education. During her subsequent follow-up visit, the patient reported improvement with no further episodes.

Case 2

A nine-year-old fourth-grade student with a history of separation anxiety, ADHD, and auditory hallucinations. He was reporting mood-congruent auditory and visual hallucinations which were thought to be attributed to anxiety. He reported seeing monsters following him and was fearful they would harm him. After an initial evaluation, he was referred to a child therapist by his psychiatrist to further explore the ongoing symptoms that were causing interference with daily living. The therapist started treatment using cognitive behavioral therapy (CBT), exposure-based therapy, and principles of dialectical behavior therapy (DBT), however, discovered that he may have also had a history of trauma over the years with witnessing his dad having a terminal illness, the parents finalizing their divorce and his mom being divorced a second time. The therapist then determined that a modality of PT informed on trauma-focused CBT (TFCBT) would be appropriate. During therapy, the dollhouse was used to reenact interactions with family members and routines, especially around bedtime. As the child engage further in the discussion, he started engaging in more direct communication and discussed his perception of challenges with the bedtime routine and encountering spaces with dim or no light. Explored how family interactions and environment could decrease distress with the anticipated events. This was helpful and so the child started to describe the different types of monsters. He reported seeing the friendly one once when he was with his mom and the unfriendly one when she was with his dad. He identified seeing more of them when he was alone and when the light was low. Through drawing, the child identified components of a “safe place” that would be then adapted into his home environment while at the same time working to increase exposure to identified worries. He started to notice the monsters were less intrusive and eventually they had become infrequent as they were “on vacation in Florida.” Upon further therapy, he opened up about feelings of feeling sad about how his dad’s terminal illness had impacted how they spend time together while also feeling undervalued by his step-mom. Although family dynamics continue to be challenging the child felt more comfortable voicing his feelings and needs to parents and also tolerating feedback in return.

Case 3

A six-year-old male child was referred by his pediatrician for trichotillomania. The child was born full-term with an immediate birth cry, and no developmental delay. The child started remaining aloof and pulled his hair after the birth of his younger sister. The child stopped interacting with his family and engaging in peer play. During the therapy session, he appeared fearful, lacked eye contact, and did not speak, he spoke in a low tone when probed. The child presented his challenges in the playroom, he picked up a doll house and four miniature dolls representing his family. Through his play, it could be found out that his parents had conflicts and he was exposed to them. NDT was chosen for him, he had three sessions in a month. This was followed by parent counseling, findings were shared. Home conflicts had reduced, and parents were mindful of not exposing the child to similar experiences. The parents reported improvement and the child stopped pulling his hair.

Case 4

A five-year-old female child was referred for school refusal. The child stopped going to school after her grandfather passed away. The child was born full-term with an immediate birth cry and no developmental delays. She was the younger of the two siblings and was over-pampered by her family. Her parents were concerned about her crying during the night, sleep disturbance, and school refusal. she often cried and vomited and got distressed when she was away from her mother. She was worried about her mother’s well-being and did not leave her sight. When she indulged in her coloring and play activities and her mother was not near, she would start crying. NDPT was commenced, as the child liked drawing, and art was used as a medium of expression. The child showed resistance when she drew her grandfather. After two sessions, the child started attending school. On follow-up parents expressed their relief as the child was sleeping better and attended school regularly.

Case 5

A nine-year-old high-functioning gymnast established care with a behavioral health therapist due to performance anxiety. Upon the initial interview, she met the criteria for generalized anxiety disorder and had strong perfectionistic tendencies and strict expectations to excel in school and gymnastics. The child became distressed when she was not able to execute skills as anticipated and will practice for many hours outside of scheduled gymnastics practice. Initially, CBT was chosen as the treatment modality with limited benefits. A later decision was made to utilize bibliotherapy as a form of PT in which selected reading material was used to guide the child to identify with the characters that had similar symptoms and experiences. this helped her normalize her feelings of worry and encouraged her to express her own story. The realization that other youth can also experience frustration through hardship was helpful. She developed an increased capacity to tolerate challenges and ultimately learned more constructive ways to respond to her anxiety. As she continued therapy, she showed improvement and increased balance of thoughts and expectations that maintained affirming personal accountability.

## Discussion

PT is a type of psychotherapy that uses play and creative activities to help children express their thoughts and emotions and work through their challenges. PT can be used to address a wide range of issues, including behavioral problems, anxiety, depression, trauma, and relationship difficulties. The therapist creates a safe and supportive environment in which the child is free to play and explore. Through play, children can communicate their experiences and feelings, learn new coping strategies, and develop positive relationships. PT has its roots in psychoanalysis and child psychology. The earliest forms of PT can be traced back to the work of Sigmund Freud and his followers, who used play and creative activities as a means of exploring a child's unconscious thoughts and feelings. In the mid-20th century, several child psychologists and psychiatrists, including Virginia Axline, Margaret Lowenfeld, and D.W. Winnicott, developed more structured approaches to PT that incorporated theories of child development and focused on helping children work through their emotional and psychological difficulties. Winnicott's approach [18] to PT emphasized the importance of the therapeutic relationship between the child and the therapist and the role of play in facilitating emotional growth and development. Winnicott believed that children use play as a means of working through their experiences, emotions, and conflicts and that the therapist's role was to provide a safe, supportive environment in which the child could play freely and express themselves. He emphasized the importance of being present and attuned to the child's play, and of not imposing one's own interpretations or expectations on the child's play. Winnicott's approach to PT is based on his concept of the “good enough” mother, which refers to the idea that the mother (or another primary caregiver) should provide the child with a supportive and nurturing environment, but also allow the child sufficient space to play and explore on their own (Table 1).

**Table 1:** History of play therapy

Author	Year	Significance
Sigmund Freud [1]	1909	The book Analysis of a Phobia in a five-year-old boy is the first documentation of the use of Play Therapy with “Little Hans”.
Melanie Klein [3]	1933	Used play as a medium for Free Association
David M. Levy [4]	1938	Used play in structured therapy called ‘Release Therapy’.
Anna Freud [5]	1946	Used play to build rapport to facilitate therapy
Carl Rogers [7]	1951	Introduced Nondirective Play Therapy (NDT)
Virginia Mae Axline [8]	1947	Expanded the work of Carl Rogers

Play is not a merely recreational activity, it has a therapeutic purpose and can be a tool for the assessment of problems, and emotional issues. PT can be used as an assessment tool and therapeutic medium to resolve the problems presented by children. Instead of trying to talk to the child and depending on his/her vocabulary become an obstacle or limitation, a medium like play can be used to let them tell their story. Freud took a more empirical approach, emphasizing winning the child’s trust and focusing on traumatic events in the child’s life, whereas Klein argued that even the very young child had unconscious impulses that might be uncovered through PT and transference.

Over the years, PT has evolved and grown, with many different approaches and techniques being developed and refined. Today, there is substantial empirical evidence supporting the effectiveness of PT in children. PT is recognized as an effective and evidence-based treatment for a wide range of psychological and emotional difficulties in children and is widely used by therapists, schools, and mental health clinics around the world. For example, a meta-analysis of 17 randomized controlled trials found that PT was effective in reducing symptoms of anxiety, depression, and behavioral problems in children [19]. Another study found that PT was effective in the reduction of levels of aggression, self-regulation, and empathy among 36 elementary school-aged children [20]. Additionally, PT has been found to be effective in improving social skills, communication skills, and self-esteem in children. The use of play and creative activities can also help children feel more relaxed and engaged in therapy, making it a valuable tool for engaging children who might be resistant to more traditional forms of therapy. PT is most used with children aged three to 12 years old. It is thought that this age range is ideal for PT as children in this stage of development tend to use play as a primary means of communication and expression. With very young children, cognitive-behavioral PT (CBPT) may be indicated, as it embeds cognitive-behavioral strategies into play-based interactions [21]. As young children may have difficulty understanding concepts in CBT, CBPT allows teaching and therapeutic work to occur in play. The primary mechanism for teaching concepts is modeling, which is effective in teaching new behaviors. Many different CBT concepts can be modeled with puppets or other toys, such as demonstrating that a puppet gets over his fear gradually the more he enters into a situation. CBPT also involves some adult administration of CBT concepts, such as scheduling activities for a withdrawn child. PT can be adapted to suit the developmental stage and individual needs of each child. For younger children, the therapist may use more structured play activities, such as sand play or dolls, to help the child express their thoughts and emotions. For older children, the therapist may use more open-ended play activities, such as drawing or storytelling, to encourage greater self-expression and exploration. PT can also be effective for teenagers and even some adults, as many people can benefit from using creative expression as a means of processing emotions and resolving psychological difficulties. However, the specific age range for which PT is most effective will depend on the individual needs and developmental stage of each person.

Like all forms of psychotherapy, PT has been the subject of criticism and debate. There are many different approaches to PT, and some critics argue that this lack of standardization can lead to inconsistencies in practice and difficulties in evaluating the effectiveness of PT. While there is a growing body of research supporting the effectiveness of PT [22-24], some critics argue that there is still a lack of well-designed, large-scale studies that demonstrate the efficacy of PT. PT often requires a substantial investment of time, and some critics argue that this can be a barrier for some families, especially those with limited resources. PT may not be appropriate for all children, particularly those with more severe or complex mental health difficulties. Some critics argue that PT may not be as effective for these children as other forms of psychotherapy. Overall, while PT has been the subject of criticism and debate, the evidence supports its effectiveness as a treatment for a wide range of psychological and emotional difficulties in children.

## Conclusions

PT is a type of psychotherapy that can be used by children who are experiencing emotional and behavioral difficulties. In spite of the fact that it is not commonly utilized, it can be beneficial, especially for young children. A wide range of issues can be addressed through PT, including behavioral problems, life transitions, trauma, and difficulties related to relationships. PT, like all forms of psychotherapy, has been subject to criticism and debate. Some critics of PT argue that the lack of standardization can result in inconsistencies in practice and difficulties in evaluating its effectiveness. PT does, however, have a growing body of research supporting its effectiveness, and modern approaches, such as CBPT, provide useful tools for treating children with depression, anxiety, trauma, and post-traumatic stress disorder (PTSD).
